# Supplementary material for: NPC1 controls TGFBR1 stability in a cholesterol transport-independent manner and promotes hepatocellular carcinoma progression
Source: Nat Commun. 2025 Jan 7;16:439. doi: 10.1038/s41467-024-55788-5 (PMC11704005; doi:10.1038/s41467-024-55788-5)
Supplement: Supplementary file 1 — Supplementary Information [file 41467_2024_55788_MOESM1_ESM.pdf]

NPC1 controls TGFBR1 stability in a cholesterol transport-independent manner and promotes hepatocellular carcinoma progression

Shuangyan Li[1,2,#], Lishan Yan[2,#], Chaoying Li[2], Lijuan Lou[2], Fengjiao Cui[2,5], Xiao Yang[2], Fuchu He[2,3,4*] and Ying Jiang[2,4*]

[1]School of Life Sciences, Tsinghua University, Beijing, China.

[2]State Key Laboratory of Medical Proteomics, National Center for Protein Sciences (Beijing), Beijing Proteome Research Center, Beijing Institute of Lifeomics, Beijing, China.

[3]Research Unit of Proteomics Dirven Cancer Precision Medicine, Chinese Academy of Medical Sciences, Beijing, China.

[4]Anhui Medical University, Hefei, China

[5]School of Basic Medicine, Qingdao University, Qingdao, China.

[#]These authors contributed equally: Shuangyan Li, Lishan Yan.

*Correspondences:

Ying Jiang: jiangying@ncpsb.org.cn; Phone/Fax: 8610-61777071

Or Fuchu He: hefc@bmi.ac.cn; Phone/Fax: 08610-61777001

Supplementary Figures and Figure legends

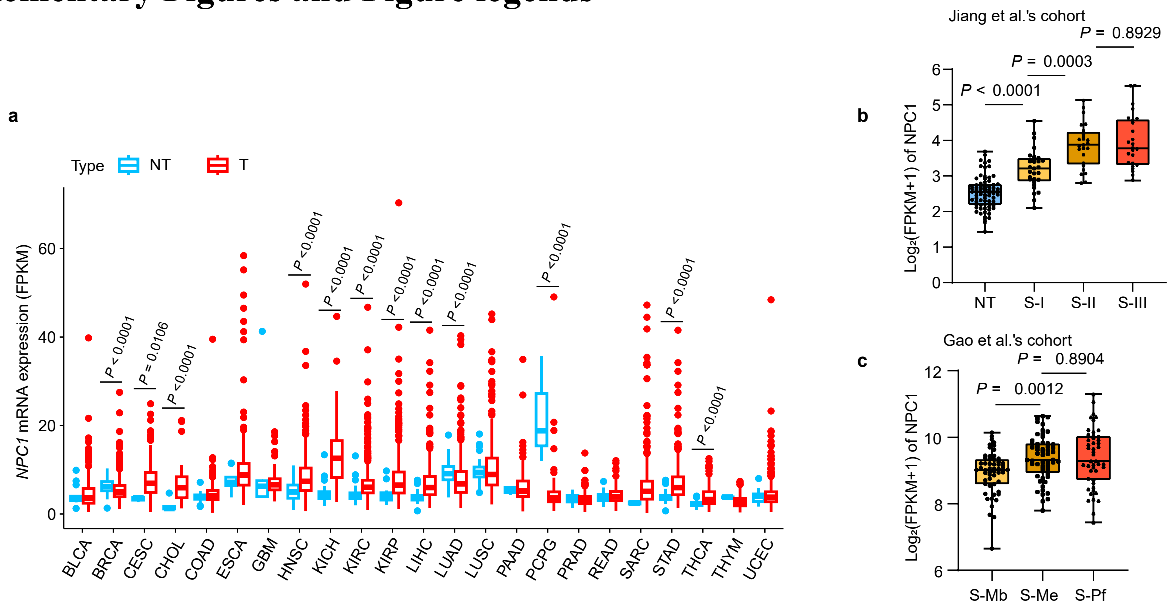

Supplementary Fig. 1 | NPC1 is upregulated in various cancers. a, *NPC1* mRNA expression between normal tissues adjacent to the tumor (NT) and tumor tissues (T) in 23 cancer types in TCGA datasets. The sample sizes are indicated in the source data. **b**, **c**, Upregulation of *NPC1* mRNA in paired non-tumor tissues (NT) and tumor tissues (T) in Jiang et al.'s cohort (**b**) (NT, n = 73; S-I, n =29; S-II, n =25; S-III n =25) and Gao et al.'s cohort (**c**) (S-Mb, n =55; S-Me, n =57; S-Pf, n =47). In the box plots, the middle bar represents the median, and the box represents the interquartile range; bars extend to 1.5× the interquartile range. Statistical significance was determined by Mann-Whitney U test (**a-c**). Source data are provided as a Source Data file.

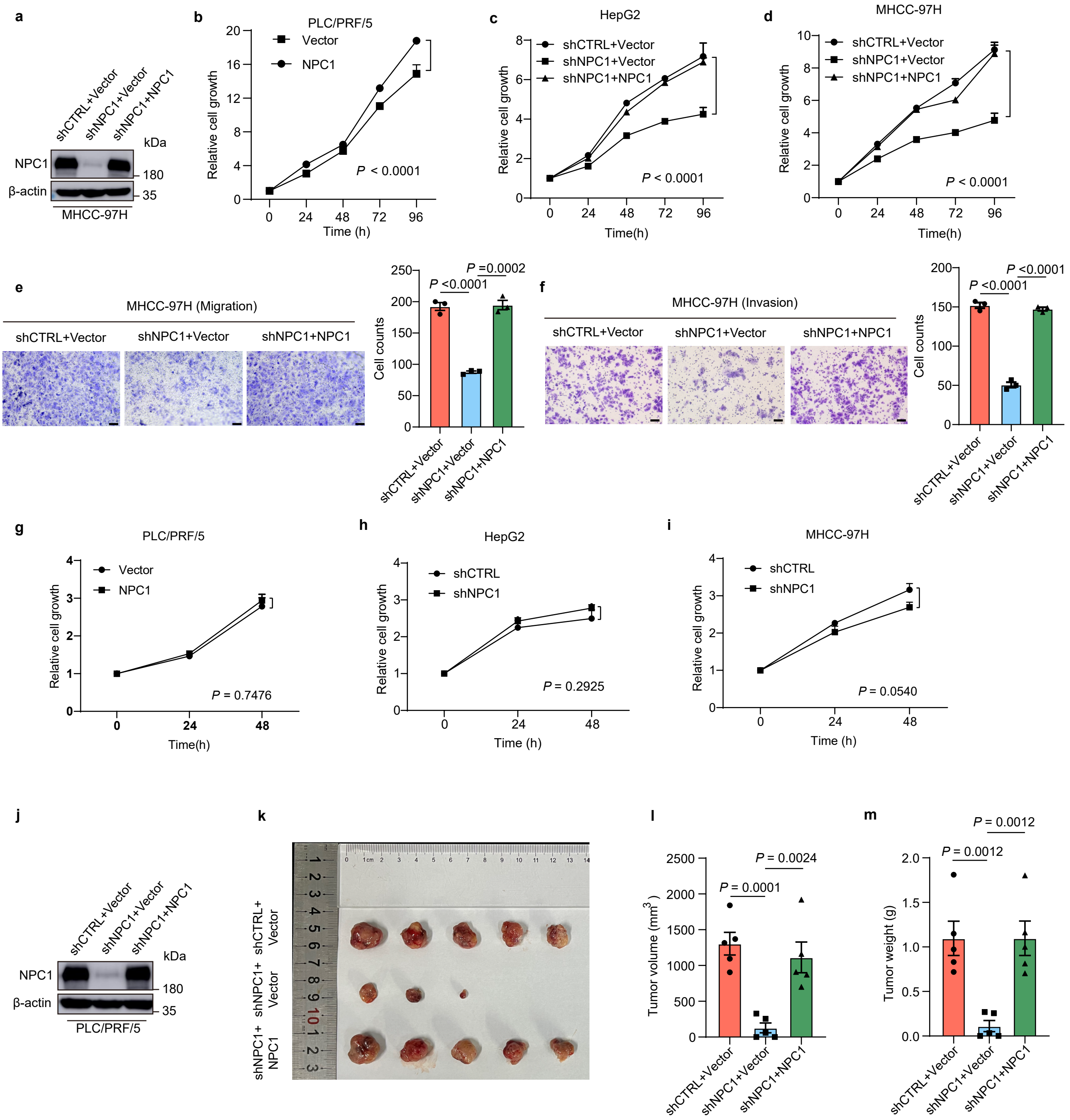

Supplementary Fig. 2 | NPC1 promotes HCC growth and metastasis. a, Confirmation of NPC1 knockdown and re-expression in HCC cells. **b-d**, Cell growth curves were measured in HCC cells with NPC1 stable overexpression (**b**) or knockdown (**c**, **d**). The relative cell number was expressed as fold change to Day 0. **e**, **f**, Transwell assay to examine the effect of NPC1 on HCC cell migration (**e**) or invasion (**f**); scale bars, 100 μm. **g-i**, NPC1 didn't have significant effect on

cell proliferation of HCC cells when they were cultured in serum free medium for 48 h. **j**-**m**, Confirmation of NPC1 knockdown and re-expression in PLC/PRF/5 cells (**j**). Photographs of xenograft tumors induced by the subcutaneous inoculation of NCG mice (n = 5 mice per group) with NPC1-knockdown PLC/PRF/5 cells with further overexpression of NPC1 (**k**). Graphs of xenograft tumor volumes (**l**), and xenograft tumor weights (**m**). Data are presented as the mean ± s.e.m. n = 3 (**b-i**) biologically independent samples. Statistical significance was determined by two-way analysis of variance (ANOVA) (**b-d**) or two-tailed unpaired Student's t-test (**e-i, l-m**). **a-j** Data were verified in three independent experiments. Source data are provided as a Source Data file.

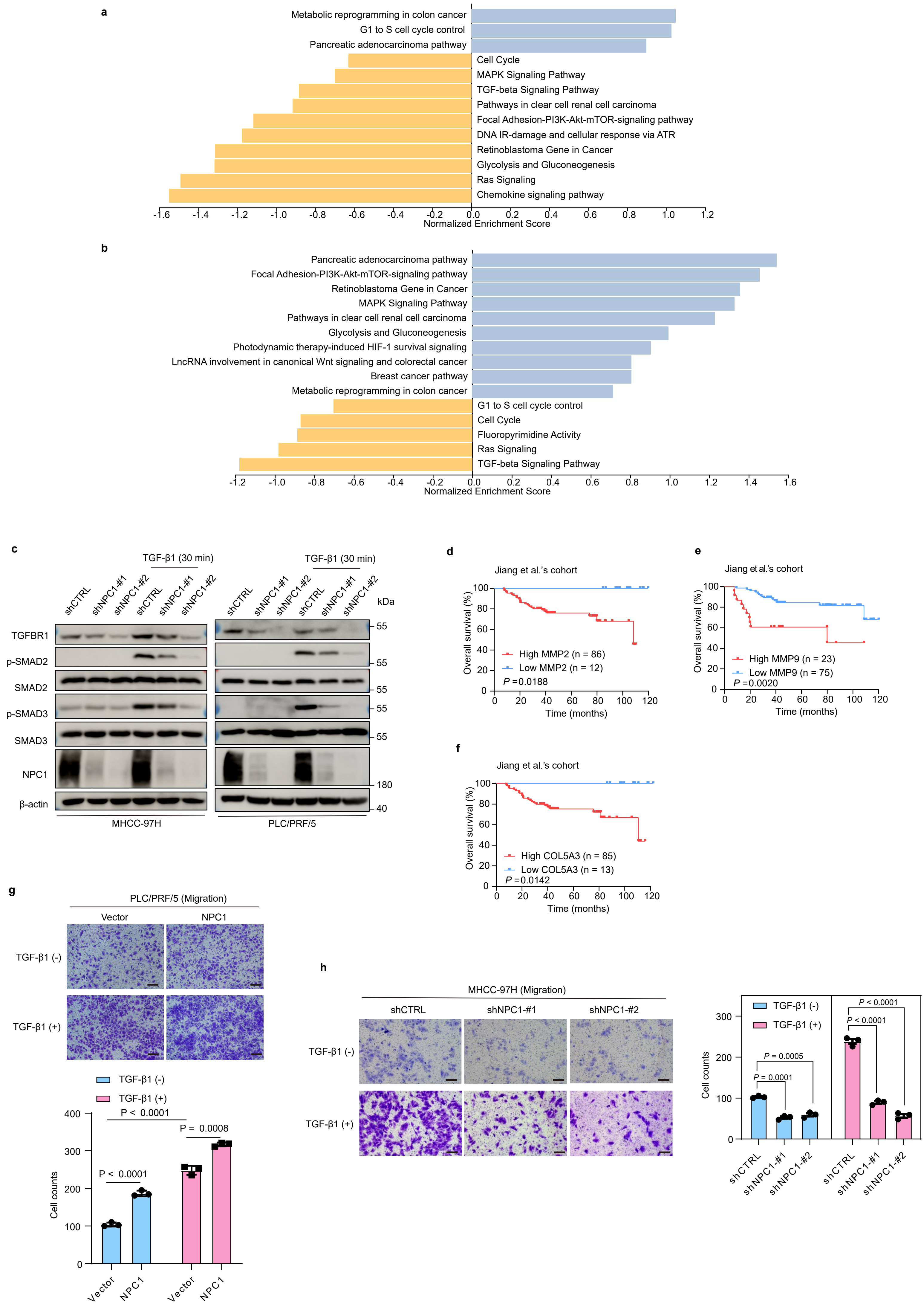
a
Metabolic reprogramming in colon cancer
G1 to S cell cycle control
Pancreatic adenocarcinoma pathway
Cell Cycle
MAPK Signaling Pathway
TGF-beta Signaling Pathway
Pathways in clear cell renal cell carcinoma
Focal Adhesion-PI3K-Akt-mTOR-signaling pathway
DNA IR-damage and cellular response via ATR
Retinoblastoma Gene in Cancer
Glycolysis and Gluconeogenesis
Ras Signaling
Chemokine signaling pathway
Normalized Enrichment Score
b
Pancreatic adenocarcinoma pathway
Focal Adhesion-PI3K-Akt-mTOR-signaling pathway
Retinoblastoma Gene in Cancer
MAPK Signaling Pathway
Pathways in clear cell renal cell carcinoma
Glycolysis and Gluconeogenesis
Photodynamic therapy-induced HIF-1 survival signaling
LncRNA involvement in canonical Wnt signaling and colorectal cancer
Breast cancer pathway
Metabolic reprogramming in colon cancer
G1 to S cell cycle control
Cell Cycle
Fluoropyrimidine Activity
Ras Signaling
TGF-beta Signaling Pathway
Normalized Enrichment Score
c
TGF-β1 (30 min)
shCTRL shNPC1-#1 shNPC1-#2 shCTRL shNPC1-#1 shNPC1-#2
kDa
TGFBR1
p-SMAD2
SMAD2
p-SMAD3
SMAD3
NPC1
β-actin
55
55
55
55
55
180
40
MHCC-97H
PLC/PRF/5
d
Jiang et al.'s cohort
Overall survival (%)
High MMP2 (n = 86)
Low MMP2 (n = 12)
P =0.0188
Time (months)
e
Jiang et al.'s cohort
Overall survival (%)
High MMP9 (n = 23)
Low MMP9 (n = 75)
P =0.0020
Time (months)
f
Jiang et al.'s cohort
Overall survival (%)
High COL5A3 (n = 85)
Low COL5A3 (n = 13)
P =0.0142
Time (months)
g
PLC/PRF/5 (Migration)
Vector
NPC1
TGF-β1 (-)
TGF-β1 (+)
TGF-β1 (-)
TGF-β1 (+)
P < 0.0001
P < 0.0001
P = 0.0008
Cell counts
Vector NPC1 Vector NPC1
h
MHCC-97H (Migration)
shCTRL
shNPC1-#1
shNPC1-#2
TGF-β1 (-)
TGF-β1 (+)
TGF-β1 (-)
TGF-β1 (+)
P < 0.0001
P < 0.0001
P = 0.0005
P = 0.0001
Cell counts
shCTRL shNPC1-#1 shNPC1-#2 shCTRL shNPC1-#1 shNPC1-#2

Supplementary Fig. 3 | NPC1 regulates TGF-β pathway. a, **b**, Gene Set Enrichment Analysis (GSEA) of different expression genes (DEGs). Pathway alterations in the PLC/PRF/5 (a) or HepG2 (**b**) cell lines after the NPC1 knockdown. Upregulated and downregulated pathways are indicated in blue (right) and yellow (left) bars. **c**, Immunoblot analysis of TGFBR1, p-SMAD2, p-SMAD3, SMAD2, SMAD3 and NPC1 expression in MHCC-97H and PLC/PRF/5 cells with NPC1 stable knockdown. **d**-**f**, Kaplan–Meier overall survival curves of individuals with high or low MMP2 (**d**), MMP9 (**e**) or COL5A3 (**f**) expression. **g**, Transwell assay was performed in NPC1-overexpression PLC/PRF/5 cells with or without TGF-β1 (10 ng/mL) treatment; scale bars, 100 μm. **h**, Transwell assay was performed in NPC1-knockdown MHCC-97H cells with or without TGF-β1 (10 ng/mL) treatment; scale bars, 100 μm. Data are presented as the mean ± s.e.m. n = 3 (**a, b, g, h**) biologically independent samples. Statistical significance was determined by two-tailed unpaired Student's t-test (**g, h**) or log-rank test (**d-f**). **a, b, g, h** Data were verified in three independent experiments. Source data are provided as a Source Data file.

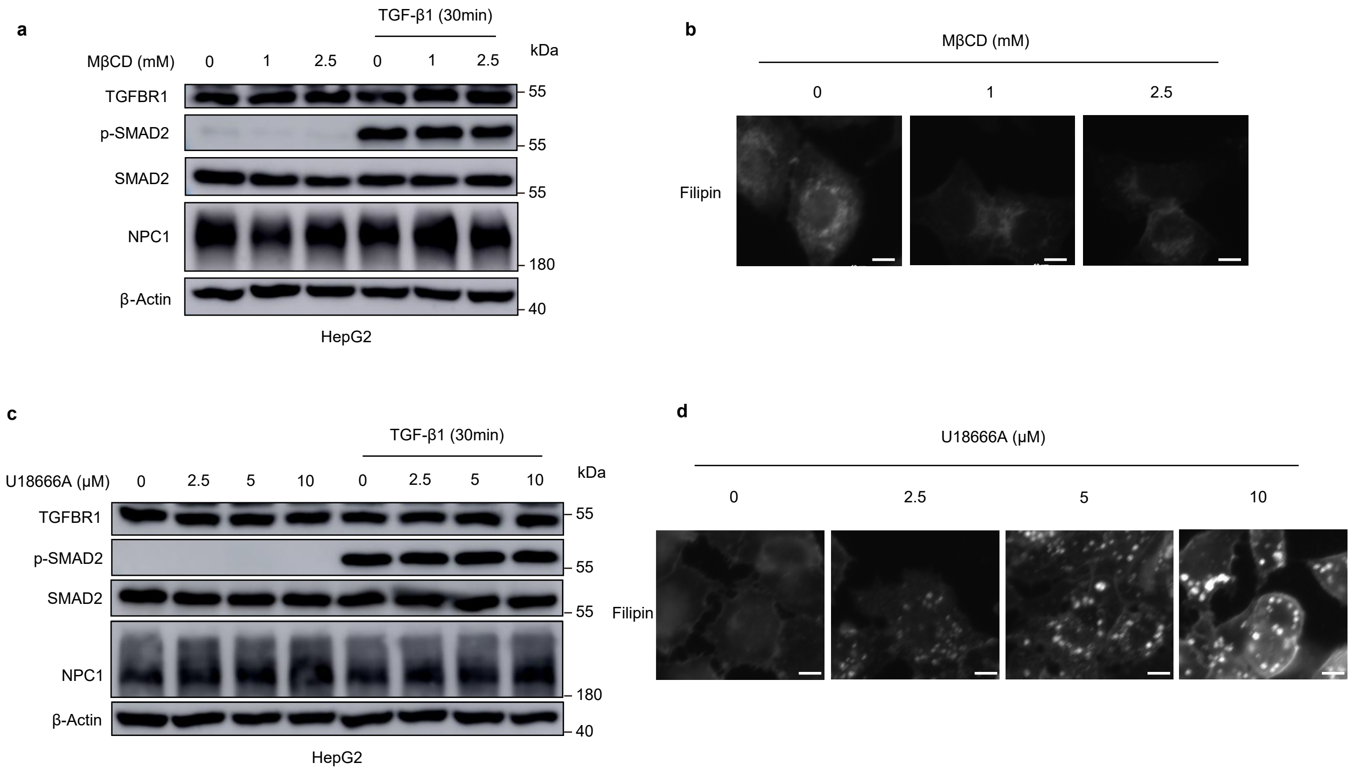

Supplementary Fig. 4 | NPC1 regulates TGF-β pathway in a cholesterol transport-independent manner. a-d, HepG2 cells were treated with MβCD (**a**) or U18666A (**c**), then analyzed by western blot to measure whole cell expression of TGFBR1 or the activity of TGF-β pathway. Cells related to (**a, c**) were fixed and stained with filipin to label free cholesterol accumulated in LE/Ly; scale bars, 10 μm (**b, d**). All experimental data were verified in three independent experiments. Source data are provided as a Source Data file.

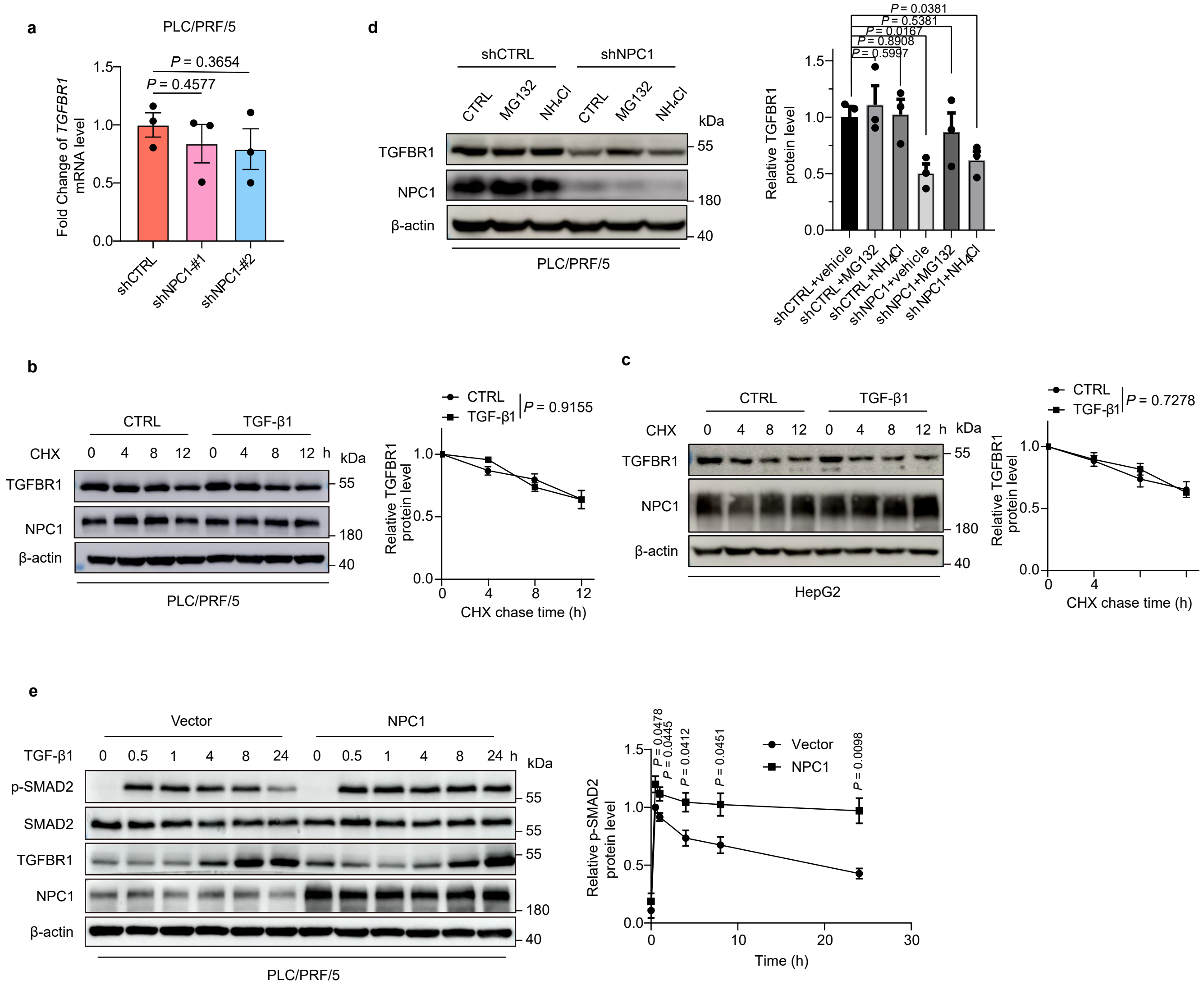

Supplementary Fig. 5 | NPC1 increases protein stability of TGFBR1 regardless of TGF-β1 treatment. a, qPCR analysis of NPC1 and TGFBR1 mRNA levels in NPC1-knockdown PLC/PRF/5 cells. **b**, **c**, PLC/PRF/5 (**b**) or HepG2 (**c**) cells were treated with CHX with or without TGF-β1 (10 ng/mL) for indicated times and then analyzed by western blot. **d**, PLC/PRF/5 cells with or without stable knockdown of NPC1 were treated with vehicle, MG132 (10 μM), or NH_4Cl (10 mM) for 12 hours. Cell lysates were subjected to immunoblot with TGFBR1 or NPC1 antibody. **e**, PLC/PRF/5 cells with stable overexpression of NPC1 were stimulated with TGF-β1 (10 ng/mL) for the times indicated. Levels of p-SMAD2, SMAD2, TGFBR1 and NPC1 were assayed by western blot. Data are presented as the mean ± s.e.m. n = 3 (**a-e**) biologically independent samples.

Statistical significance was determined by two-tailed unpaired Student's t-test (**a, d, e**) or two-way analysis of variance (ANOVA) (**b**, **c**). All experimental data were verified in three independent experiments. Source data are provided as a Source Data file.

8

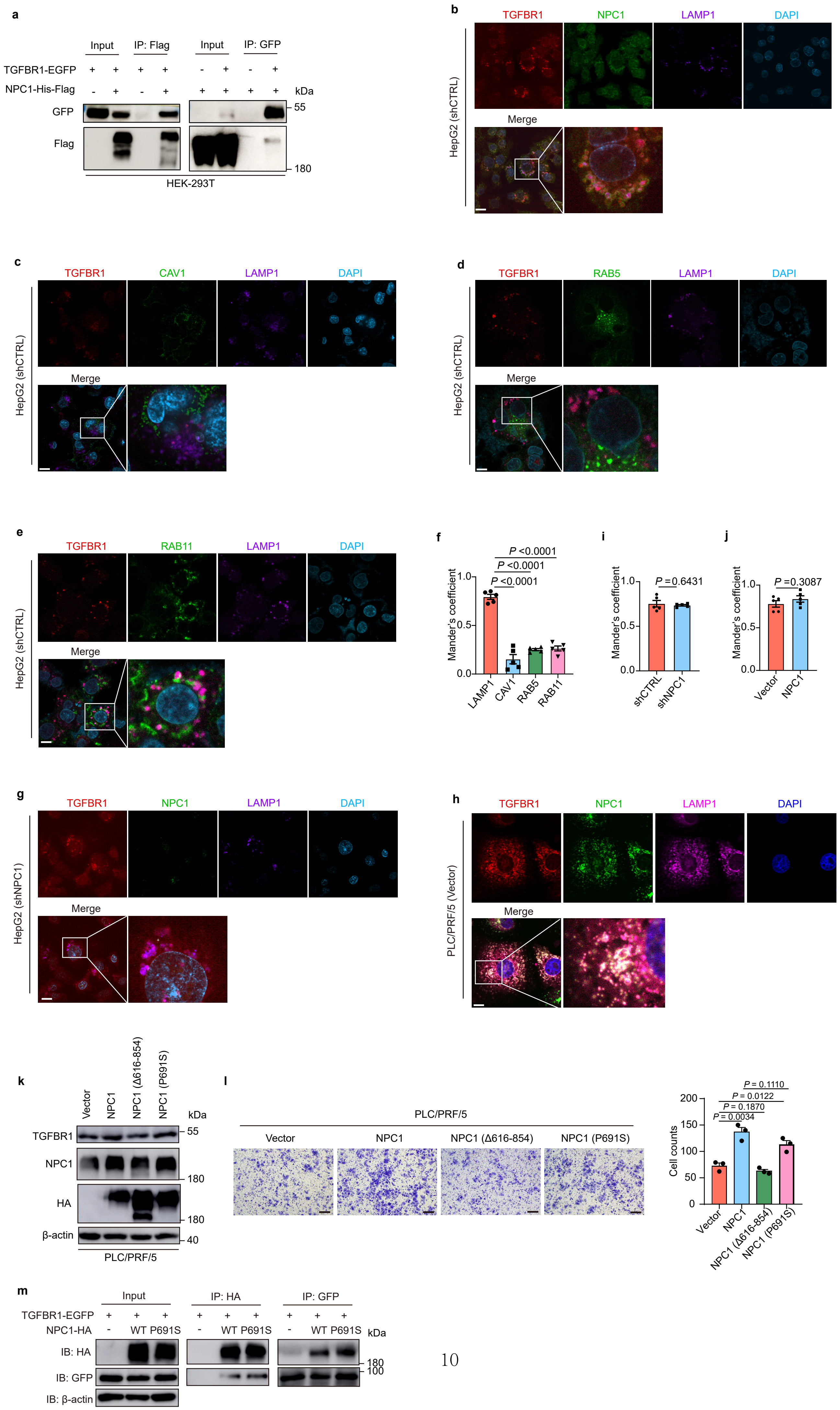

a
Input
IP: Flag
Input
IP: GFP
TGFBR1-EGFP
NPC1-His-Flag
kDa
GFP
Flag
55
180
HEK-293T
b
TGFBR1
NPC1
LAMP1
DAPI
HepG2 (shCTRL)
Merge
c
TGFBR1
CAV1
LAMP1
DAPI
HepG2 (shCTRL)
Merge
d
TGFBR1
RAB5
LAMP1
DAPI
HepG2 (shCTRL)
Merge
e
TGFBR1
RAB11
LAMP1
DAPI
HepG2 (shCTRL)
Merge
f
P <0.0001
P <0.0001
P <0.0001
Mander's coefficient
LAMP1
CAV1
RAB5
RAB11
i
P =0.6431
Mander's coefficient
shCTRL
shNPC1
j
P =0.3087
Mander's coefficient
Vector
NPC1
g
TGFBR1
NPC1
LAMP1
DAPI
HepG2 (shNPC1)
Merge
h
TGFBR1
NPC1
LAMP1
DAPI
PLC/PRF/5 (Vector)
Merge
k
Vector
NPC1
NPC1 (Δ616-854)
NPC1 (P691S)
kDa
TGFBR1
NPC1
HA
β-actin
55
180
180
40
PLC/PRF/5
l
PLC/PRF/5
Vector
NPC1
NPC1 (Δ616-854)
NPC1 (P691S)
P = 0.1110
P = 0.0122
P = 0.1870
P = 0.0034
Cell counts
Vector
NPC1
NPC1 (Δ616-854)
NPC1 (P691S)
m
Input
IP: HA
IP: GFP
TGFBR1-EGFP
NPC1-HA
WT P691S
kDa
IB: HA
IB: GFP
IB: β-actin
180
100

Supplementary Fig. 6 | NPC1 interacts with TGFBR1. a, The lysates of HEK-293T transfected with indicated constructs were subjected to immunoprecipitation with anti-Flag (or GFP) antibody. The immunoprecipitates were then immunoblotted with anti-GFP (or Flag) antibody. **b**, HepG2 cells stably expressing TGFBR1-mCherry-His were immunostained with antibodies against NPC1 and LAMP1 to determine the colocalization among TGFBR1, NPC1 and LAMP1 in HepG2 cells. **c**, HepG2 cells stably expressing TGFBR1-mCherry-His and Caveolin-1 (CAV1)-Myc were immunostained with antibodies against Myc and LAMP1 to determine the colocalization among TGFBR1, CAV1 and LAMP1 in HepG2 cells. **d-e**, HepG2 cells stably expressing TGFBR1-mCherry-His were immunostained with antibodies against RAB5 (**d**) /RAB11 (**e**) and LAMP1 to determine the colocalization among TGFBR1, RAB5/ RAB11 and LAMP1 in HepG2 cells. **f**, Quantification of the colocalization between these four proteins (LAMP1, CAV1, RAB5 and RAB11) and TGFBR1 using the Mander's coefficient. **g,** NPC1-knockdown HepG2 cells stably expressing TGFBR1-mCherry-His were immunostained with antibodies against NPC1 and LAMP1 to determine the colocalization among TGFBR1, NPC1 and LAMP1 in HepG2 cells **h,** PLC/PRF/5 cells stably overexpressing TGFBR1-mCherry-His and HA-tag were immunostained with antibodies against NPC1 and LAMP1 to determine the colocalization among TGFBR1, NPC1 and LAMP1 in PLC/PRF/5 cells. Representative images from three independent experiments (**b-e, g-h**) are shown; scale bars, 10 μm. **i-j**, Quantification of the colocalization between TGFBR1 and

LAMP1 using the Mander's coefficient in HepG2 cells with NPC1 stable knockdown (**i**) or PLC/PRF/5 cells with NPC1 stable overexpression (**j**). **k**, Immunoblot analysis of TGFBR1 and NPC1 expression in PLC/PRF/5 cells with overexpression of NPC1, NPC1 (Δ616-854), or NPC1 (P691S). **l**, Transwell assay was performed in cells related to (**k**) (n = 3 biologically independent samples); scale bars, 100 μm. **m**, NPC1-HA or NPC1 (P691S)-HA stable overexpression PLC/PRF/5 cells transfected with TGFBR1-EGFP plasmid were subjected to immunoprecipitation with anti-HA or anti-GFP magnetic beads. The lysates and immunoprecipitates were then blotted. Data are presented as the mean ± s.e.m. Statistical significance was determined by two-tailed unpaired Student's t-test (**f, i, j, l**). All experimental data were verified in three independent experiments. Source data are provided as a Source Data file.

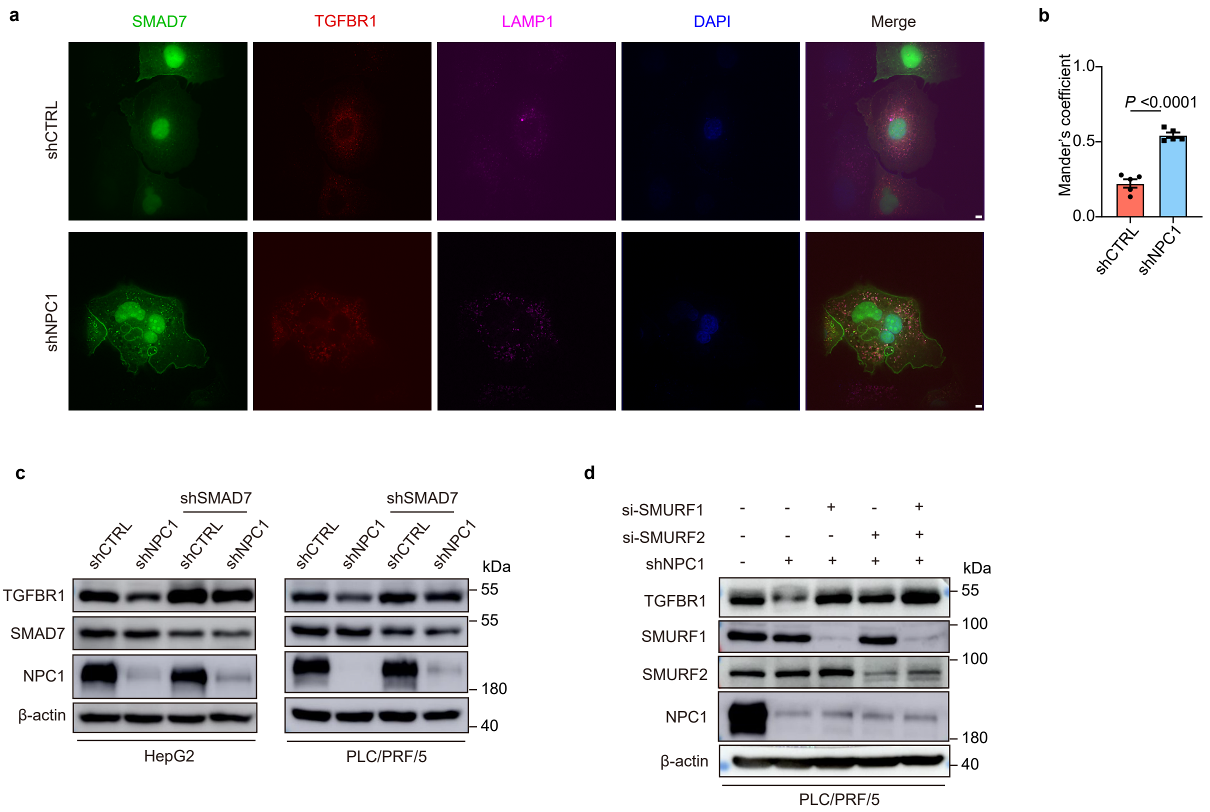

Supplementary Fig. 7 | NPC1 regulates TGFBR1 in a SMAD7-dependent manner. a, PLC/PRF/5 cells stably expressing TGFBR1-mCherry-His and SMAD7-EGFP were immunostained with antibodies against LAMP1 and then imaged by Structure Illumination Microscopy to determine the colocalization among TGFBR1, SMAD7 and LAMP1 in PLC/PRF/5 cells with NPC1 stable knockdown. The images were collected through the Polar-SIM system. **b**, Quantification of the colocalization between TGFBR1 and SMAD7 using the Mander's coefficient in PLC/PRF/5 cells with NPC1 stable knockdown. **c**, Immunoblot analysis of TGFBR1, SMAD7 and NPC1 expression in NPC1-knockdown HepG2 and PLC/PRF/5 cells with or without knockdown of SMAD7. **d**, Immunoblot analysis of TGFBR1, SMURF1, SMURF2 and NPC1 expression in NPC1-knockdown PLC/PRF/5 cells were transfected with specific siRNAs

targeting SMURF1 or SMURF2. Representative images from three independent experiments (**a**) are shown; scale bars, 10 μm. Data are presented as the mean ± s.e.m. Statistical significance was determined by two-tailed unpaired Student's t-test (**b**). All experimental data were verified in three independent experiments. Source data are provided as a Source Data file.

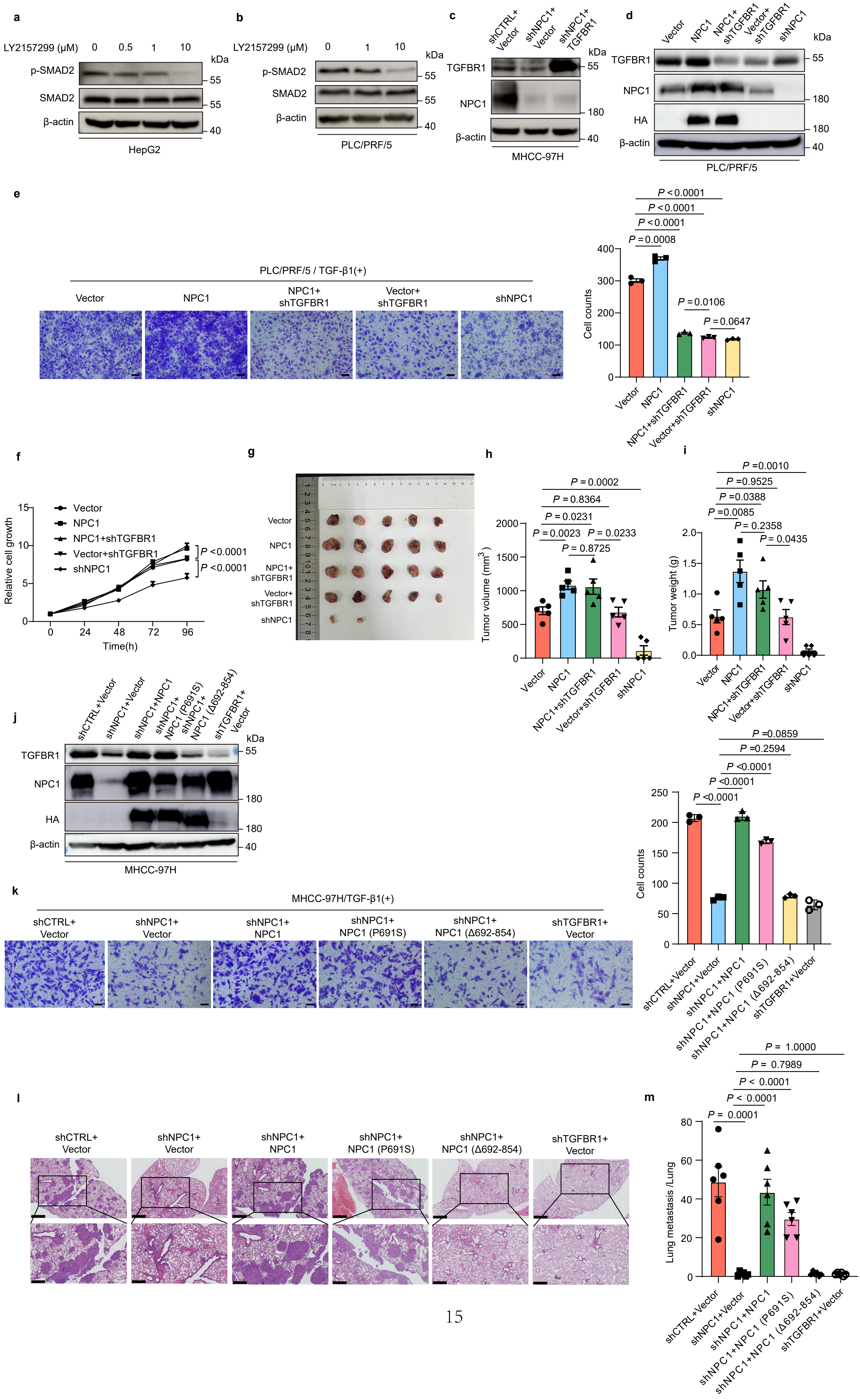

a
LY2157299 (μM) 0 0.5 1 10
p-SMAD2
SMAD2
β-actin
HepG2
b
PLC/PRF/5
c
shCTRL+Vector
shNPC1+Vector
shNPC1+TGFBR1
TGFBR1
NPC1
MHCC-97H
d
PLC/PRF/5
e
PLC/PRF/5 / TGF-β1(+)
Vector
NPC1
NPC1+shTGFBR1
Vector+shTGFBR1
shNPC1
Cell counts
f
Relative cell growth
Time(h)
g
h
Tumor volume (mm3)
i
Tumor weight (g)
j
HA
k
MHCC-97H/TGF-β1(+)
shNPC1+NPC1 (P691S)
shNPC1+NPC1 (Δ692-854)
shTGFBR1+Vector
l
m
Lung metastasis /Lung

Supplementary Fig. 8 | TGFBR1 is crucial for NPC1-mediated promotion of HCC progression. a, **b**, HepG2 (**a**) and PLC/PRF/5 (**b**) cells with or without LY2157299 treatment for 16 h were pretreated with TGF-β1 (10 ng/mL) for 30 min before collection. Cell lysates were subjected to immunoblot with p-SMAD2 or SMAD2 antibody. **c**, Immunoblot analysis of TGFBR1 and NPC1 expression in NPC1-knockdown MHCC-97H cells with or without TGFBR1 overexpression **d**, Immunoblot analysis of TGFBR1 and NPC1 expression in PLC/PRF/5 cells with stable overexpression or knockdown of indicated genes. **e**, Transwell assay was performed in cells related to (**d**); scale bars, 100 μm. **f**, Cell growth curves were measured in indicated cells. **g-i**, Photographs of xenograft tumors induced by the subcutaneous inoculation of NCG mice (n = 5 mice per group) in indicated groups (**g**). Graphs of xenograft tumor volumes (**h**), and xenograft tumor weights (**i**). **j**, Immunoblot analysis of TGFBR1 and NPC1 expression in MHCC-97H cells with stable overexpression or knockdown of indicated genes. **k**, Transwell assay was performed in cells related to (**j**); scale bars, 100 μm. **l-m**, 1×10^6 Luciferase-expressing MHCC-97H cells were injected into NCG mice by tail vein. The mice were euthanized 8 weeks later by a cervical dislocation. Representative H&E staining images of lung tissues are shown; scale bars, 1mm; insets: twofold magnification; scale bars, 500 μm (**l**). The incidence of lung metastasis in mice (**m**) (n = 6 mice per group). Data are presented as the mean ± s.e.m. n = 3 (**e, f, k**) biologically independent samples. Statistical significance was determined by two-tailed unpaired Student's t-test (**e, h, i, k, m**) or two-way

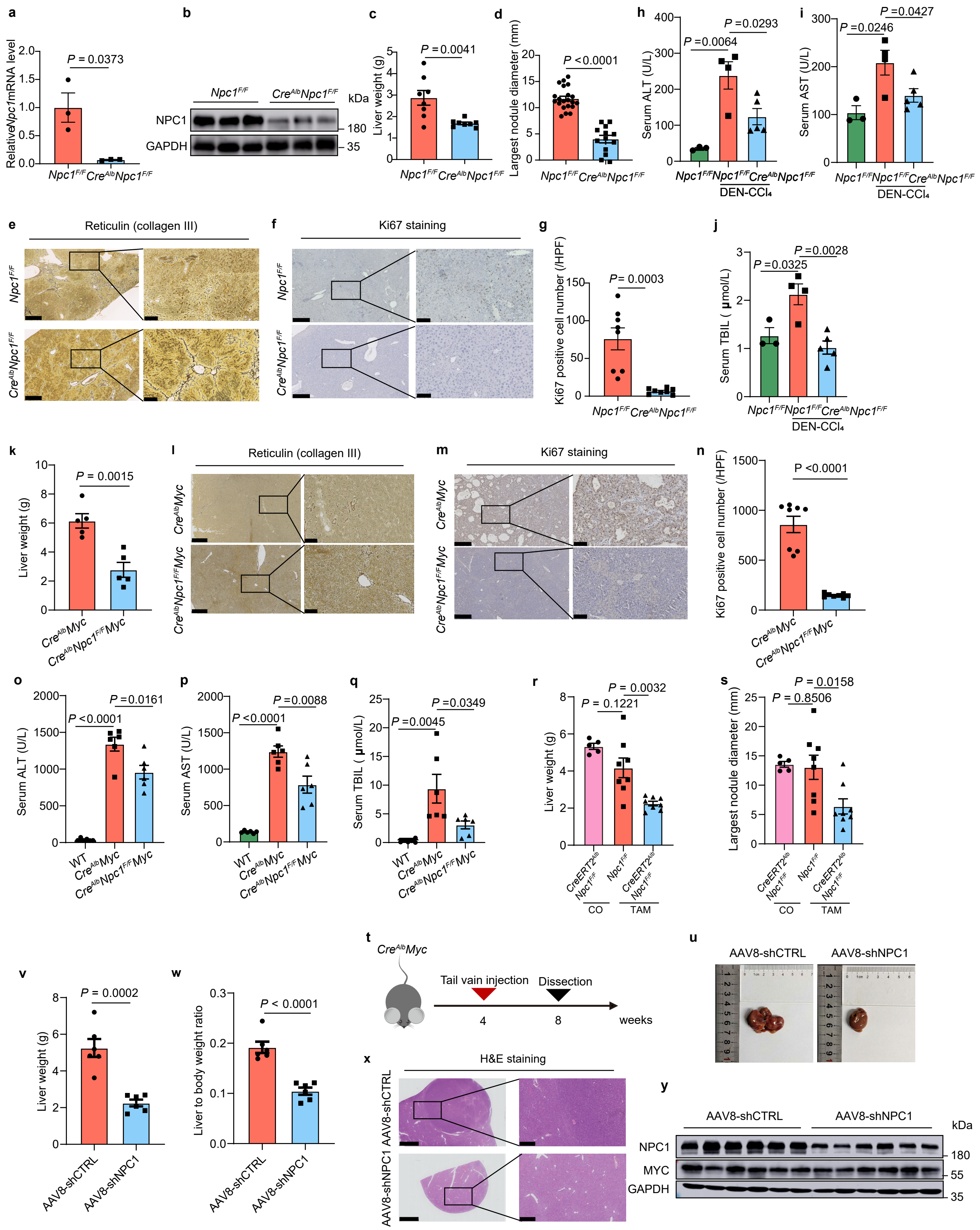

Supplementary Fig. 9 | Hepatic NPC1 deficiency inhibits HCC tumorigenesis

a, *Npc1* mRNA expression in $Npc1^{F/F}$ and $Cre^{Alb}Npc1^{F/F}$ mouse HCC tissues (n = 3 mice per group). **b**, NPC1 protein expression in $Npc1^{F/F}$ and $Cre^{Alb}Npc1^{F/F}$ mouse HCC tissues (n = 3 mice per group). **c, d**, liver weight (n = 8 mice) (**c**), diameters of the largest nodules (n = 21 mice in $Npc1^{F/F}$ and n = 13 mice in $Cre^{Alb}Npc1^{F/F}$ groups) (**d**) of the indicated mice. **e**, Representative reticulin staining images of the indicated mouse livers. Scale bars: left panels, 500 µm; right panels, 100 µm. **f**, Representative IHC staining images of Ki67 in the indicated mouse livers. Scale bars: left panels, 500 µm; right panels, 100 µm. **g**, Quantification of Ki67 staining in the indicated mouse livers (n = 8 mice). **h-j**, Analysis of serum ALT (**h**), AST (**i**) and TBIL (**j**) levels in normal $Npc1^{F/F}$ (n = 3), $Npc1^{F/F}$ (n = 4) and $Cre^{Alb}Npc1^{F/F}$ (n = 5) mice with DEN-CCl_4 induced HCC. **k,** liver weight of $Cre^{Alb}Myc$ and $Cre^{Alb}Npc1^{F/F}Myc$ mice (n = 5 mice). **l**, Representative reticulin staining images of the indicated mouse livers. Scale bars: left panels, 500 µm; right panels, 100 µm. **m**, Representative IHC staining images of Ki67 in the indicated mouse livers. Scale bars: left panels, 500 µm; right panels 100 µm. **n**, Quantification of Ki67 staining in the indicated mouse livers (n = 8 mice).**o-q**, Analysis of serum ALT (**o**), AST (**p**) and TBIL (**q**) levels in WT, $Cre^{Alb}Myc$ and $Cre^{Alb}Npc1^{F/F}Myc$ mice (n = 6 mice). **r, s**, liver weight (**r**), diameters of the largest nodules(**s**) of $Npc1^{F/F}$ mice treated with coil (CO) (n = 5 mice), $CreERT2^{Alb}Npc1^{F/F}$ mice treated with coil (CO) or tamoxifen (TAM) (n = 8 mice). **t**, Schematic view of the treatment plan using AAV serotype 8 (AAV8).
