# Supplementary material for: NPC1 controls TGFBR1 stability in a cholesterol transport-independent manner and promotes hepatocellular carcinoma progression
Source: Nat Commun. 2025 Jan 7;16:439. doi: 10.1038/s41467-024-55788-5 (PMC11704005; doi:10.1038/s41467-024-55788-5)
Supplement: Supplementary file 2 — Description of Additional Supplementary Files [file 41467_2024_55788_MOESM2_ESM.pdf]

Description of Additional supplementary files

Supplementary Data 1

Description: Tissue microarray details and clinical information

Supplementary Data 2

Description: Antibodies

Supplementary Data 3

Description: Oligonucleotide sequences of shRNA and siRNA

Supplementary Data 4

Description: Recombinant DNA

Supplementary Data 5

Description: Nucleotide sequences of primers used for quantitative real-time PCR
