# Supplementary material for: NPC1 controls TGFBR1 stability in a cholesterol transport-independent manner and promotes hepatocellular carcinoma progression
Source: Nat Commun. 2025 Jan 7;16:439. doi: 10.1038/s41467-024-55788-5 (PMC11704005; doi:10.1038/s41467-024-55788-5)
Supplement: Supplementary file 8 — Reporting Summary [file 41467_2024_55788_MOESM8_ESM.pdf]

nature portfolio

Corresponding author(s): Fuchu He and Ying Jiang

Last updated by author(s): Nov 14, 2024

Reporting Summary

Nature Portfolio wishes to improve the reproducibility of the work that we publish. This form provides structure for consistency and transparency in reporting. For further information on Nature Portfolio policies, see our Editorial Policies and the Editorial Policy Checklist.

Statistics

For all statistical analyses, confirm that the following items are present in the figure legend, table legend, main text, or Methods section.

n/a	Confirmed	
☐	☒	The exact sample size (*n*) for each experimental group/condition, given as a discrete number and unit of measurement
☐	☒	A statement on whether measurements were taken from distinct samples or whether the same sample was measured repeatedly
☐	☒	The statistical test(s) used AND whether they are one- or two-sided<br>*Only common tests should be described solely by name; describe more complex techniques in the Methods section.*
☒	☐	A description of all covariates tested
☐	☒	A description of any assumptions or corrections, such as tests of normality and adjustment for multiple comparisons
☐	☒	A full description of the statistical parameters including central tendency (e.g. means) or other basic estimates (e.g. regression coefficient) AND variation (e.g. standard deviation) or associated estimates of uncertainty (e.g. confidence intervals)
☐	☒	For null hypothesis testing, the test statistic (e.g. *F*, *t*, *r*) with confidence intervals, effect sizes, degrees of freedom and *P* value noted<br>*Give P values as exact values whenever suitable.*
☒	☐	For Bayesian analysis, information on the choice of priors and Markov chain Monte Carlo settings
☒	☐	For hierarchical and complex designs, identification of the appropriate level for tests and full reporting of outcomes
☐	☒	Estimates of effect sizes (e.g. Cohen's *d*, Pearson's *r*), indicating how they were calculated

Our web collection on statistics for biologists contains articles on many of the points above.

Software and code

Policy information about availability of computer code

Data collection	The mass spectrometry data were collected with the Xcalibur (version 3.0.63; Thermo Fisher Scientific).
Data analysis	Raw MS files were analyzed with MaxQuant (version 1.6.1.0) under default parameters against the human UniProt database (version 20180705). Further difference analysis and functional enrichment analysis was performed using Perseus software and WebGestalt web tool.All statistical and sample size information are shown in the figures, figure legends and source data. Data distribution was assumed to be normal, but this was not formally tested. The allocation in animal experiments was random. Data collection and analysis were not performed blind to the conditions of the experiments, except for IHC score analysis. Each experiment was repeated independently with similar results unless indicated in the figure legends. Quantitative data are presented as mean ± s.e.m.. Statistical analyses were performed in GraphPad Prism 8. Two-tailed unpaired Student’s t-test, Mann-Whitney U test and two-way analysis of variance (ANOVA) were used to calculate P values. Kaplan–Meier curves were used to depict survival function from lifetime data for human patients using the log-rank test. Exact P values are indicated in all figures, and a P value less than 0.05 was considered statistically significant.

For manuscripts utilizing custom algorithms or software that are central to the research but not yet described in published literature, software must be made available to editors and reviewers. We strongly encourage code deposition in a community repository (e.g. GitHub). See the Nature Portfolio guidelines for submitting code & software for further information.

Data

Policy information about availability of data

All manuscripts must include a data availability statement. This statement should provide the following information, where applicable:

- Accession codes, unique identifiers, or web links for publicly available datasets
- A description of any restrictions on data availability
- For clinical datasets or third party data, please ensure that the statement adheres to our policy

The mass spectrometry proteomics data have been deposited to the ProteomeXchange Consortium (http://proteomecentral.proteomexchange.org) via the iProX partner repository with the dataset identifier PXD046018 (https://www.iprox.cn//page/project.html?id=IPX0007268000).
The public data reanalyzed in our study were from TCGA datasets (https://www.cancer.gov/about-nci/organization/ccg/research/structural-genomics/tcga). The HCC proteome data that support the findings of this study were from the iProX database "Proteomics identifies new therapeutic targets of early-stage hepatocellular carcinoma" and are accessible through iProX accession number IPX0000937000 (https://www.iprox.cn//page/project.html?id=IPX0000937000). The data of transcriptome sequencing and proteome from "Integrated Proteogenomic Characterization of HBVRelated Hepatocellular Carcinoma" can be viewed in NODE (https://www.biosino.org/node) by pasting the accession (OEP000321) into the text search box or through the URL: https://www. biosino.org/node/project/detail/OEP000321.

Research involving human participants, their data, or biological material

Policy information about studies with human participants or human data. See also policy information about sex, gender (identity/presentation), and sexual orientation and race, ethnicity and racism.

Reporting on sex and gender	*Use the terms sex (biological attribute) and gender (shaped by social and cultural circumstances) carefully in order to avoid confusing both terms. Indicate if findings apply to only one sex or gender; describe whether sex and gender were considered in study design; whether sex and/or gender was determined based on self-reporting or assigned and methods used. Provide in the source data disaggregated sex and gender data, where this information has been collected, and if consent has been obtained for sharing of individual-level data; provide overall numbers in this Reporting Summary. Please state if this information has not been collected. Report sex- and gender-based analyses where performed, justify reasons for lack of sex- and gender-based analysis.*
Reporting on race, ethnicity, or other socially relevant groupings	*Please specify the socially constructed or socially relevant categorization variable(s) used in your manuscript and explain why they were used. Please note that such variables should not be used as proxies for other socially constructed/relevant variables (for example, race or ethnicity should not be used as a proxy for socioeconomic status). Provide clear definitions of the relevant terms used, how they were provided (by the participants/respondents, the researchers, or third parties), and the method(s) used to classify people into the different categories (e.g. self-report, census or administrative data, social media data, etc.) Please provide details about how you controlled for confounding variables in your analyses.*
Population characteristics	*Describe the covariate-relevant population characteristics of the human research participants (e.g. age, genotypic information, past and current diagnosis and treatment categories). If you filled out the behavioural & social sciences study design questions and have nothing to add here, write "See above."*
Recruitment	*Describe how participants were recruited. Outline any potential self-selection bias or other biases that may be present and how these are likely to impact results.*
Ethics oversight	*Identify the organization(s) that approved the study protocol.*

Note that full information on the approval of the study protocol must also be provided in the manuscript.

Field-specific reporting

Please select the one below that is the best fit for your research. If you are not sure, read the appropriate sections before making your selection.

☒ Life sciences ☐ Behavioural & social sciences ☐ Ecological, evolutionary & environmental sciences

For a reference copy of the document with all sections, see nature.com/documents/nr-reporting-summary-flat.pdf

Life sciences study design

All studies must disclose on these points even when the disclosure is negative.

Sample size	In vitro functional assays including CCK-8, transwell migration and invasion were performed in three independent experiments. The NOD SCID mouse experiments were performed with $n \geq 6$ biological independent samples for each group. The NCG mouse experiments were performed with $n \geq 5$ biological independent samples for each group. The C57BL/6J mouse experiments were performed with $n \geq 3$ biological independent samples for each group. Where possible, sample sizes were chosen based on established protocols in generally-accepted criteria in the scientific community.
Data exclusions	No data was excluded for analysis.

Replication	Repetitive biologically independent experiments were performed to validate the consistency of results. Biologically independent experiments and sample sizes with consistent results are indicated in the figure legends.
Randomization	The allocation in mouse experiments is random.
Blinding	Although we could not fulfill blinding in our assays, we performed these assays with fixed parameters in a same experiment (i.e., data from control and experimental groups were all collected in the same parameters). In light of this, we have largely avoided the influence of human eyes and subjectivity on the conclusion in this study.

Reporting for specific materials, systems and methods

We require information from authors about some types of materials, experimental systems and methods used in many studies. Here, indicate whether each material, system or method listed is relevant to your study. If you are not sure if a list item applies to your research, read the appropriate section before selecting a response.

Materials & experimental systems

n/a	Involved in the study
☐	☒ Antibodies
☐	☒ Eukaryotic cell lines
☒	☐ Palaeontology and archaeology
☐	☒ Animals and other organisms
☒	☐ Clinical data
☒	☐ Dual use research of concern
☒	☐ Plants

Methods

n/a	Involved in the study
☒	☐ ChIP-seq
☒	☐ Flow cytometry
☒	☐ MRI-based neuroimaging

Antibodies

Antibodies used

anti-NPC1 (clone JB87-33, rabbit mAb (IHC), invitrogen, Cat# MA5-34694, 1:3000 dilution),
anti-Ki-67 (Rabbit polyclonal Ab, Abcam, Cat# Ab15580, 1:3000 dilution),
anti-EpCAM (clone E6V8Y, rabbit mAb, Cell Signaling, Cat# 93790, 1:300 dilution),
anti-GRP-78 (BiP) (clone C50B12, rabbit mAb, Cell Signaling, Cat# 3177, 1:200 dilution),
anti-Keratin19 (rabbit polyclonal Ab, Abcam, Cat# Ab15463, 1:100 dilution),
anti-NPC1 (Rabbit monoclonal Ab (IP), Abcam, Cat# Ab224268, 4ug/sample),
anti-NPC1 (Rabbit monoclonal Ab (IB), Abcam, Cat# Ab134113, 1:10000 dilution),
anti-TGFBR1 (Rabbit monoclonal Ab (IB), Abcam, Cat# Ab235578, 1:1000 dilution),
anti-TGFBR1 (Rabbit monoclonal Ab (IP), Abcam, Cat# Ab235578, 4ug/sample),
Rabbit IgG (IP),Cell Signaling, Cat#2729, 4ug/sample),
anti-TGFBR1 (Rat monoclonal Ab (IHC), R&D Systems, Cat# MAB5871, 1:2000 dilution),
anti-LAMP1 (clone D4O1S, Mouse mAb, Cell Signaling, Cat#15665, 1:100 dilution),
anti-RAB5 (Rabbit monoclonal Ab, Abcam, Cat# Ab218624, 1:100 dilution),
anti-RAB11 (clone D4F5, Rabbit mAb, Cell Signaling, Cat#5589, 1:50 dilution),
anti-c-Myc/N-Myc (clone D3N8F, Rabbit mAb, Cell Signaling, Cat# 13987, 1:1000 dilution),
anti-Phospho-SMAD2 (Ser465/467) (clone 138D4, Rabbit mAb, Cell Signaling, Cat# 3108S, 1:1000 dilution),
anti-SMAD2 (clone D43B4, Rabbit mAb, Cell Signaling, Cat# 12584S, 1:1000 dilution),
anti-Phospho-SMAD3 (Ser423/425) (clone C25A9, Rabbit mAb, Cell Signaling, Cat# 9520S, 1:1000 dilution),
anti-SMAD3 (clone C67H9, Rabbit mAb, Cell Signaling, Cat# 9523S, 1:1000 dilution),
anti-LDLR (Rabbit monoclonal Ab, Abcam , Cat# Ab52818, 1:1000 dilution),
anti-SMAD7 (clone 293039, Monoclonal Mouse IgG2B, R&D Systems, Cat# MAB2029, 1:1000 dilution),
anti-SMURF1 (Mouse monoclonal Ab, Abcam, Cat# Ab57573, 1:1000 dilution),
anti-SMURF2 (Rabbit monoclonal Ab , Abcam, Cat# Ab53316, 1:1000 dilution),
anti-K48-linkage Specific Polyubiquitin (clone D9D5, Rabbit mAb, Cell Signaling, Cat# 8081S, 1:1000 dilution),
anti-K63-linkage Specific Polyubiquitin (clone D7A11, Rabbit mAb, Cell Signaling, Cat# 5621S, 1:1000 dilution),
anti-Multi Ubiquitin (Mouse mAb, MBL, Cat# D058-3, 1:1000 dilution),
anti-mCherry tag (Mouse mAb, Solarbio, Cat# K200015M, 1:1000 dilution),
anti-GFP (Mouse mAb, MBL , Cat# MO48-3, 1:1000 dilution),
anti-DDDDK-tag (Mouse mAb, MBL, Cat# M185-3L, 1:5000 dilution),
anti-HA-Tag (clone 6E2, Mouse mAb (IB) , Cell Signaling, Cat# 2367S, 1:1000 dilution),
anti-HA-Tag (clone C29F4, Rabbit mAb (IF), Cell Signaling, Cat# 3724S, 1:100 dilution),
anti-His-Tag (Mouse Monoclonal Ab, EASYBIO, Cat# BE2019, 1:1000 dilution),
anti-Myc-tag (Mouse Monoclonal Ab (IB), MBL, Cat# M047-3, 1:1000 dilution),
anti-Myc-tag (clone 71D10, Rabbit mAb (IF), Cell Signaling, Cat# 2278, 1:50 dilution),
anti-β-actin (clone OTI1, Mouse mAb, ZSGB-Bio, Cat# TA-09, 1:1000 dilution),
anti-GAPDH (Rabbit Polyclonal Ab, Proteintech, Cat# 10494-1-AP, 1:1000 dilution),
anti-SREBP2 (Rabbit polyclonal Ab, Abcam, Cat# Ab30682, 1:1000 dilution),
anti-HMGCR (Rabbit polyclonal Ab, Invitrogen, Cat# PA5-37367, 1:1000 dilution),
anti-HMGCS1 (clone D1Q9D, Rabbit mAb, Cell Signaling, Cat# 42201S, 1:1000 dilution),
anti-ABCG5(Rabbit Polyclonal Ab, Proteintech, Cat# 27722-1-AP, 1:1000 dilution),
anti-ABCG8(Rabbit Polyclonal Ab, Proteintech, Cat# 24453-1-AP, 1:1000 dilution).

Validation

Rabbit monoclonal anti-NPC1 (Cat# MA5-34694) was validated by the manufacture (more information is available at https://www.thermofisher.cn/cn/zh/antibody/product/NPC1-Antibody-clone-JB87-33-Recombinant-Monoclonal/MA5-34694)
Rabbit polyclonal anti-Ki-67 (Cat# Ab15580) has been previously used (PMID: 39353535 and validated by the manufacture (more information is available at https://www.abcam.cn/products%2fprimary-antibodies%2fki67-antibody-ab15580.html)
Rabbit monoclonal anti-EpCAM (Cat# 93790) has been previously used (PMID: 35440604) and validated by the manufacture (more information is available at https://www.cellsignal.com/products/primary-antibodies/epcam-e6v8y-xp-rabbit-mab/93790)
Rabbit monoclonal anti-GRP-78 (BiP) (Cat# 3177) has been previously used (PMID: 37743418) and validated by the manufacture (more information is available at https://www.cellsignal.com/products/primary-antibodies/bip-c50b12-rabbit-mab/3177)
Rabbit polyclonal anti-Keratin19 (Cat# Ab15463) has been previously used (PMID: 35922425) and validated by the manufacture (more information is available at https://www.abcam.com/products/primary-antibodies/cytokeratin-19-antibody-ab15463.html)
Rabbit monoclonal anti-NPC1 (Cat# Ab224268) has been previously used (PMID: 30902833) and validated by the manufacture (more information is available at https://www.abcam.com/products/primary-antibodies/niemann-pick-c1-antibody-epr5209-bsa-and-azide-free-ab224268.html)
Rabbit monoclonal anti-NPC1 (Cat# Ab134113) has been previously used (PMID: 35480314) and validated by the manufacture (more information is available at https://www.abcam.com/products/primary-antibodies/niemann-pick-c1-antibody-epr5209-ab134113.html)
Rabbit monoclonal anti-TGFBR1 (Cat# Ab235578) has been previously used (PMID: 36263180) and validated by the manufacture (more information is available at https://www.abcam.com/products/primary-antibodies/tgf-beta-receptor-i-antibody-epr20923-13-ab235578.html)
Rabbit IgG (Cat# 2729) has been previously used (PMID: 39271675) and validated by the manufacture (more information is available at https://www.cellsignal.com/products/primary-antibodies/normal-rabbit-igg/2729)
Rat monoclonal anti-TGFBR1 (Cat# MAB5871) has been previously used (PMID: 26692002) and validated by the manufacture (https://www.rndsystems.com/cn/products/human-mouse-tgf-beta-ri-alk-5-antibody-141231_mab5871#product-citations)
Mouse monoclonal anti-LAMP1(Cat#15665) has been previously used (PMID: 37659079) and validated by the manufacture (more information is available at https://www.cellsignal.cn/products/primary-antibodies/lamp1-d4o1s-mouse-mab/15665
Rabbit monoclonal anti-RAB5(Cat# Ab218624) has been previously used (PMID: 37949856) and validated by the manufacture (more information is available at https://www.abcam.cn/products/primary-antibodies/rab5-antibody-epr21801-early-endosome-marker-ab218624.html?productWallTab=Abreviews)
Rabbit monoclonal anti-RAB11(Cat# 5589) has been previously used (PMID: 37141099) and validated by the manufacture (more information is available at https://www.cellsignal.cn/products/primary-antibodies/rab11-d4f5-xp-rabbit-mab/5589)
Rabbit monoclonal anti-c-Myc/N-Myc(Cat# 13987) has been previously used (PMID: 38040699) and validated by the manufacture (more information is available at https://www.cellsignal.cn/products/primary-antibodies/c-myc-n-myc-d3n8f-rabbit-mab/13987)
Rabbit monoclonal anti-Phospho-SMAD2 (Ser465/467) (Cat# 3108S) has been previously used (PMID: 37799511) and validated by the manufacture (more information is available at https://www.cellsignal.com/products/primary-antibodies/phospho-smad2-ser465-467-138d4-rabbit-mab/3108)
Rabbit monoclonal SMAD2 (Cat# 12584S) has been previously used (PMID: 37034204) and validated by the manufacture (more information is available at https://www.cellsignal.com/products/antibody-conjugates/smad2-d43b4-xp-rabbit-mab-biotinylated/12584)
Rabbit monoclonal anti-Phospho-SMAD3 (Ser423/425) (Cat# 9520S) has been previously used (PMID: 37749092) and validated by the manufacture (more information is available at https://www.cellsignal.com/products/primary-antibodies/phospho-smad3-ser423-425-c25a9-rabbit-mab/9520)
Rabbit monoclonal anti-SMAD3 (Cat# 9523S) has been previously used (PMID: 37800598) and validated by the manufacture (more information is available at https://www.cellsignal.com/products/primary-antibodies/smad3-c67h9-rabbit-mab/9523)
Mouse monoclonal anti-SMAD7 (Cat# MAB2029) has been previously used (PMID: 35538070) and validated by the manufacture (more information is available at https://www.rndsystems.com/cn/products/human-mouse-rat-smad7 antibody-293039_mab2029#product-citations)
Mouse monoclonal anti-SMURF1 (Cat# Ab57573) has been previously used (PMID: 36370851) and validated by the manufacture (more information is available at https://www.abcam.com/products/primary-antibodies/smurf1-antibody-1d7-ab57573.html)
Rabbit monoclonal anti-SMURF2 (Cat# Ab53316) has been previously used (PMID: 33097595) and validated by the manufacture (more information is available at https://www.abcam.com/products/primary-antibodies/smurf-2-antibody-ep629y3-ab53316.html)
Rabbit monoclonal anti-K48-linkage Specific Polyubiquitin (Cat# 8081S) has been previously used (PMID: 37641865) and validated by the manufacture (more information is available at https://www.cellsignal.com/products/primary-antibodies/k48-linkage-specific-polyubiquitin-d9d5-rabbit-mab/8081)
Rabbit monoclonal anti-K63-linkage Specific Polyubiquitin (Cat# 5621S) has been previously used (PMID: 37248861) and validated by the manufacture (more information is available at https://www.cellsignal.com/products/primary-antibodies/k63-linkage-specific-polyubiquitin-d7a11-rabbit-mab/5621)
Mouse monoclonal anti-Multi Ubiquitin (Cat# D058-3) has been previously used (PMID: 16230742) and validated by the manufacture (more information is available at https://www.mblintl.com/products/d058-3/)
Mouse Monoclonal anti-mCherry (Cat# K200015M) has been validated by the manufacture (https://www.solarbio.com/goods-64424.html#related-products-and-xglw)
Mouse Monoclonal anti-GFP (Cat# MO48-3) has been previously used (PMID:32641624) and validated by the manufacture (https://ruo.mbl.co.jp/bio/dtl/A/?pcd=M048-3)
Mouse Monoclonal anti-DDDDK (Cat# M185-3L) has been previously used (PMID:26928300) and validated by the manufacture (more information is available at https://www.mblbio.com/bio/g/dtl/A/?pcd=M185-3L)
Mouse Monoclonal anti-HA (Cat# 2367S) has been previously used (PMID:38012141) and validated by the manufacture (more information is available at https://www.cellsignal.cn/products/primary-antibodies/ha-tag-6e2-mouse-mab/2367#pdpCiteABCitations)
Rabbit Monoclonal anti-HA (Cat# 3724S) has been previously used (PMID:38664376) and validated by the manufacture (more information is available at https://www.cellsignal.cn/products/primary-antibodies/ha-tag-c29f4-rabbit-mab/3724)
Mouse Monoclonal anti-His (Cat# BE2019) has been previously used (PMID: 31932609) and validated by the manufacture (more information is available at http://bioeasytech.com/product/2387.html?goods_id=4305)
Mouse Monoclonal anti-Myc(Cat# M047-3) has been previously used (PMID:20159986) and validated by the manufacture (more information ishttps://ruo.mbl.co.jp/bio/e/dtl/A/index.html?pcd=M047-3#u-pub)
Rabbit Monoclonal anti-Myc(Cat# 2278) has been previously used (PMID:38608030) and validated by the manufacture (more information is available at https://www.cellsignal.cn/products/primary-antibodies/myc-tag-71d10-rabbit-mab/2278)
Mouse Monoclonal Anti-β actin(Cat# TA-09) has been validated by the manufacture (more information is available at http://

www.zsbio.com/product/TA-09)
Rabbit Polyclonal anti-GAPDH(Cat# 10494-1-AP) has been previously used (PMID: 33658717) and validated by the manufacture (more information is available at https://www.ptgcn.com/Products/GAPDH-Antibody-10494-1-AP.htm#publications)

Eukaryotic cell lines

Policy information about cell lines and Sex and Gender in Research

Cell line source(s)	Human HCC cell lines (MHCC-97H, HepG2, PLC/RPF/5) and HEK-293T cells were used in this study. MHCC-97H cells were obtained from Liver Cancer Institute, Zhongshan Hospital, Fudan University. HepG2(ATCC, HB-8065), HEK-293T (SCSP-502) and PLC/RPF/5 (TCHu119) cells purchased from Stem Cell Bank/Stem Cell Core Facility, SIBCB, CAS.
Authentication	Using short tandem repeat (STR) profiling method.
Mycoplasma contamination	All the cells were tested negative for mycoplasma.
Commonly misidentified lines (See ICLAC register)	No commonly misidentified cell lines were used.

Animals and other research organisms

Policy information about studies involving animals; ARRIVE guidelines recommended for reporting animal research, and Sex and Gender in Research

Laboratory animals	Mice were maintained on a 12-h light-dark cycle (08:00-20:00 light, 20:00-08:00dark)in a temperature-controlled (23 ± 2 °C), relative humidity (40-50%) andpathogen-free facility with free access to food and water. All animals were maintained on normal chow diet (SPF-F02-003) until treatment. Age- and sex-matched mice were used for experiments. Liver-specific conditional Npc1-knockout mice (CreAlbNpc1F/F, 2–4 months old, 3 males and 4 females), Npc1F/F (2–4 months old,8 males and 7 females)and CreERT2Alb mice(6-8 weeks old,2males) in the C57BL/6J background were made by Cyagen with CRISPR-Cas9. Non-obese diabetic/severe-combined immunodeficiency (NOD/SCID) mice (5 weeks old, Female) were purchased from Beijing Vital River Laboratory Animal Technology. NCG mice (5 weeks old, male) were purchased from GemPharmatech Co. Ltd (Nanjing, China). H11-CAG-LSL-Myc mice (Cat. NO. NM-KI-00039,2–4 months old, 2 males and 2 females) were purchased from Shanghai Model Organisms. The animal care and experimental protocols were approved by the Institutional Animal Care and Use Committee (IACUC) of the National Center for Protein Sciences, Beijing, China (NCPSB).
Wild animals	Our study did not include wild animals.
Reporting on sex	Sex was not considered in this study.
Field-collected samples	Our study did not involve samples collected from field.
Ethics oversight	All animal assays were approved by Institutional Animal Care and Use Committee (IACUC) of the National Center for Protein Sciences, Beijing, China (NCPSB) (No: NCPSB-20230912-55MT-R1).

Note that full information on the approval of the study protocol must also be provided in the manuscript.

Plants

Seed stocks	*Report on the source of all seed stocks or other plant material used. If applicable, state the seed stock centre and catalogue number. If plant specimens were collected from the field, describe the collection location, date and sampling procedures.*
Novel plant genotypes	*Describe the methods by which all novel plant genotypes were produced. This includes those generated by transgenic approaches, gene editing, chemical/radiation-based mutagenesis and hybridization. For transgenic lines, describe the transformation method, the number of independent lines analyzed and the generation upon which experiments were performed. For gene-edited lines, describe the editor used, the endogenous sequence targeted for editing, the targeting guide RNA sequence (if applicable) and how the editor was applied.*
Authentication	*Describe any authentication procedures for each seed stock used or novel genotype generated. Describe any experiments used to assess the effect of a mutation and, where applicable, how potential secondary effects (e.g. second site T-DNA insertions, mosiacism, off-target gene editing) were examined.*
